# Beyond symptoms: How mental health competencies shape everyday well-being among psychiatric patients

**DOI:** 10.1192/j.eurpsy.2025.695

**Published:** 2025-08-26

**Authors:** F. Pongracz, V. Zabo, D. Erat, A. Vargha, A. Vincze, J. Harangozo, M. Ivancsics, J. Farkas, G. Balogh, J. Bognar, E. Nagy, X. Gonda

**Affiliations:** 1 Faculty of Education and Psychology, Institute of Psychology; 2 Doctoral School of Psychology, Eotvos Lorand University; 3 Faculty of Medicine, Institute of Behavioural Sciences, Semmelweis University, Budapest; 4 Department of Sociology, University of Pecs, Pecs; 5 Person- and Family-Oriented Health Science Research Group, Faculty of Humanities and Social Sciences, Institute of Psychology, Károli Gáspár University of the Reformed Church in Hungary; 6 Nyiro Gyula Hospital, National Institute of Psychiatry and Addictology; 7 Community Psychiatry Centre, Semmelweis University; 8 Awakenings Foundation; 9 Department of Pediatrics; 10 Department of Psychiatry and Psychotherapy, Semmelweis University, Budapest, Hungary

## Abstract

**Introduction:**

Just as our bodies have immune systems to defend against harmful biological agents, our souls also need psychological “immune competencies” to cope with stress. These competencies include effective emotional, psychological, social and spiritual functioning, resilience, creative and executive efficiency, self-regulation and savoring, the ability to enjoy positive experiences.

**Objectives:**

The aim of the present study was to investigate whether the mental health competencies, the symptoms of mental disorder, or the interaction of the two have a stronger predictive power on subjective well-being among Hungarian adult psychiatric patients.

**Methods:**

The psychiatric sample of 129 patients (44 men, 85 women) was recruited in a cross-sectional design in four Hungarian health care facilities. Participants completed the Symptom Checklist-90-Revised, the Mental Health Test and six well-being questionnaires.

**Results:**

Mental health competencies are stronger predictors of the three indicators of well-being (β = 0.61; 0.79; 0.51 p < 0.05) than mental disorder symptoms (β = 0.17; 0.12; 0.25, p < 0.05). Including both mental health competencies and mental disorder symptoms in a regression model more accurately predicts indicators of well-being (BIC = 310; 359.7; 170; AIC = 289; 337.3; 148.3; R^2^ = 0.74; 0.52; 0.58, p < 0.05) than either the effect of the two separately (BIC = 310.3; 365.4; 170.2; AIC = 291.1; 345.8; 151.1; R^2^ = 0.73; 0.48; 0.56, p > 0.05) or the effect of their interaction (BIC = 314.9; 363.6; 173.6; AIC = 290.3; 338.4; 149.1; R^2^ = 0.74; 0.52; 0.57, p > 0.05). Mental health competencies were positively (B = 0.88; 1.64; 0.54, p < 0.05) while mental disorder symptoms were negatively (B = -0.50; -0.28; -0,17, p < 0.05) related to indicators of subjective well-being.

**Conclusions:**

The results underscore the potential of mental health competencies as protective factors that can enhance well-being and restore daily functioning even in the presence of mental disorder symptoms.

**Disclosure of Interest:**

None Declared

